# Analysis of the capsular bend in posterior capsular opacification using anterior segment optical coherence tomography

**DOI:** 10.1007/s10792-023-02897-7

**Published:** 2023-10-28

**Authors:** Asmaa M Gamal El-Deen

**Affiliations:** Present Address: Department of Ophthalmology, Faculty of Medicine (for Girls), Al-Azhar University, Cairo, Egypt

**Keywords:** Posterior capsular opacification, Capsular bend, Anterior segment optical coherence tomography, PCO score

## Abstract

**Purpose:**

To investigate the link between the capsular bend and the morphological types and characteristics of posterior capsular opacification (PCO) using anterior segment optical coherence tomography.

**Methods:**

Thirty eyes with PCO were examined, and three types of PCO were identified: pearl, fibrosis, and mixed. We assessed anterior capsular overlap, intraocular lens-capsule adhesion, and capsular bending. In addition to measuring the intraocular lens-posterior capsule distance and capsule bending angle (CBA), the PCO parameters (area, density, and score at 6-, 5-, and 3-mm intraocular lens optic regions) were recorded. The associations between capsular bend and PCO type and characteristics were investigated. A control group of 12 eyes without PCO was used to compare the study variables.

**Results:**

With p values greater than 0.001, there was a statistically significant difference in the mean PCO area and score at the 6-, 5-, and 3-mm optic zones in different PCO types, with the pearl type having the highest value, followed by the mixed type, and finally the fibrosis type. The PCO group had a significantly higher mean CBA than the control group (*P* = 0.001). CBA was positively related to intraocular lens-posterior capsule distance, PCO area, and PCO score at the 6-, 5-, and 3-mm zones (*P* = 0.001). The receiver operating characteristic curve's cut-off point for CBA was 96.85° when comparing PCO cases to controls. Partial overlap and incomplete adhesion were statistically more common in the PCO eyes than in the control (*P* = 0.001, 0.003, respectively).

**Conclusion:**

PCO types and CBA have a strong relationship with PCO score and intraocular lens-posterior capsule space. In PCO's eyes, CBA has a cut-off value of 96.85°.

**Supplementary Information:**

The online version contains supplementary material available at 10.1007/s10792-023-02897-7.

## Background

Posterior capsular opacification (PCO), also known as 'secondary cataract' or 'after cataract', develops over the clear posterior capsule (PC) months to years after an uneventful cataract surgery. During cataract surgery, lens epithelial cells (LEC) grow and proliferate on the capsule. These cells migrate to the PC, where they obstruct the central visual axis, resulting in decreased vision [1].

There are two types of PCO: fibrous and pearl PCO. A combination of both is occasionally observed. Fibrous PCO is thought to be caused by LEC lining the anterior capsule. Clinically, it appears as wrinkling on the PC capsule at the site of anterior–posterior capsule fusion [2]. Pearl, or proliferative PCO, is caused by LEC lining the pre-equatorial zone. It reveals clusters of swollen, opacified, differentiated LEC known as bladder or Wedl cells upon examination [3]. A buildup of fibroblast-like cells and fibrous components in fibrous PCO tissue, as well as the spherical lentoid structures of Elschnig's pearls, is visible under electron microscopy on the inner surface of the PC behind the optic portion of the intraocular lens (IOL) [4]. Lens-like tissue can regenerate in the capsule's peripheral bag, known as the Soemmering's ring [5].

Interactions between the IOL and capsular bag have always piqued the interest of cataract surgeons owing to their role in the pathogenesis of PCO formation. Various tools have been developed over time to investigate the dynamics of the IOL-PC space [2, 6]. The space configuration between the anterior capsule, PC, and IOL is represented by a capsular bend [7]. The previous research has shown that capsular bend caused by an IOL with a sharp optic edge can help prevent PCO [8, 9].

Existing PCO evaluation methods include a subjective scoring system and an objective system, both of which are based on automated analysis of retro-illumination images. The Scheimpflug image is an objective method for directly determining the density of the PCO. These methods primarily score PCO severity by multiplying the PCO fraction by density, which can be graded subjectively [10].

Optical coherence tomography (OCT) allows high-resolution cross-sectional imaging of tissues [11] and can thus be used for further PCO characterization. PCO intensity and thickness in patients after cataract surgery can be estimated using anterior segment OCT (AS-OCT). Because OCT has no observer bias [12] and allows for a more objective analysis than slit-lamp images, it has the potential to become an additional tool in PCO grading systems. However, automatic image processing is required for these purposes to speed up image analysis and allow the measurement of the IOL-PC distance over the selected area [13].

## Materials and methods

This observational study was conducted between March 2022 and October 2022 at our hospital. The study was conducted in accordance with the Helsinki Declaration of 2013, and approval from the Research Ethical Committee of our hospital was obtained prior to the start of the study. Before providing written informed consent, all participants were provided with a thorough explanation of the study. Thirty eyes from patients with PCO and 12 eyes from participants without substantial PCO (the control group) were included in the study. The phacoemulsification procedure with manual capsulorhexis and implantation of a single-piece acrylic IOL (EYECRYI^®^ 600, Biotech, India) went smoothly for every participant. Patients with intraoperative complications (e.g., PC rupture, zonolysis) or postoperative complications (e.g., posterior synechia, bag-IOL subluxation), sulcus-fixated IOLs, prior ocular surgery or trauma, history of corneal pathology, glaucoma, pseudoexfoliation syndrome, uveitis, posterior segment pathology, and insufficient media clarity were excluded.

We collected demographic and clinical data, including age, gender, interval from cataract surgery, best corrected visual acuity (BCVA), intraocular pressure, and slit-lamp and funduscopic examinations. PCO was graded using the following method described by Aslam and Patton [14]:

- Grade 0: Nothing visible
- Grade 1: Visible but does not extend to the edge of the IOL
- Grade 2: At the edge of IOL
- Grade 3: Inside the IOL's edge, but the visual axis is clear
- Grade 4: gaining access to the visual axis

AS-OCT (RTVue-100 OCT, Optovue Inc., Fremont, CA, USA) was used to capture anterior segment images. A corneal anterior module lens was installed on the detection probe after pupil dilation. Participants focused their other eye on the front red indicator light. Using the cornea-cross line mode, images of the lens capsule on the vertical and horizontal meridians were captured while focusing on the IOL plane. Images were analyzed using the Image-J software. For the purpose of detecting pixels, images were calibrated using known distances in both the vertical and horizontal directions.

**Fig. 1:**
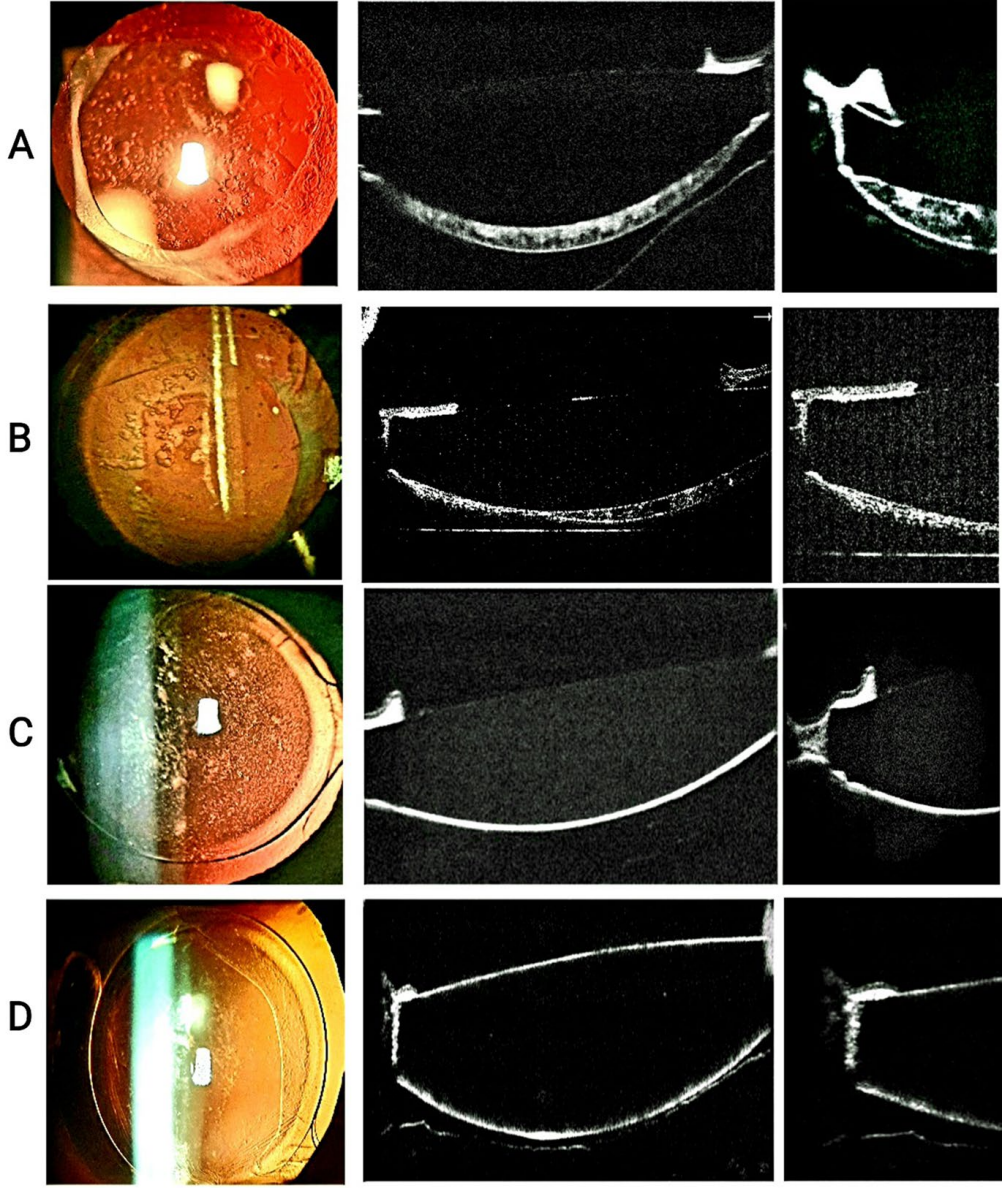
Representative images of different PCO types from left to right: the first column shows retroillumination images, the second demonstrates RTVue-100 OCT B scan images, and the third shows the Capsular bend; **A** pseudophakic eye with pearl-type PCO; **B** Mixed-type; **C** Fibrosis-type; **D** Without clinically significant PCO(control eye)

The PCO types were classified clinically and by using AS-OCT images into three groups: fibrosis, pearl, and mixed (Fig. 1). In some cases, the gap between the IOL and PC was filled with clear or milky fluid**.** These cases are consistent with Miyake et al. [15] for late-postoperative capsular block syndrome. We did not include these cases in the present study.

The area, density, and score of opacification at the 6-, 5-, and 3- mm optic zones were measured. IOL-PC distance and capsule bending angle (CBA) were also measured. The values of the vertical and horizontal meridians were averaged for further analysis (Supplementary Fig. 1).

The PCO score was calculated by multiplying the PCO area by the mean density after adjusting for the influence of the IOL scattered light intensity. The average light scatter density of the IOL's central 5 mm by 0.25 mm area was measured and subtracted from the average PCO density to remove the influence of the IOL on the intensity of scattered light [16].

**Fig. 2:**
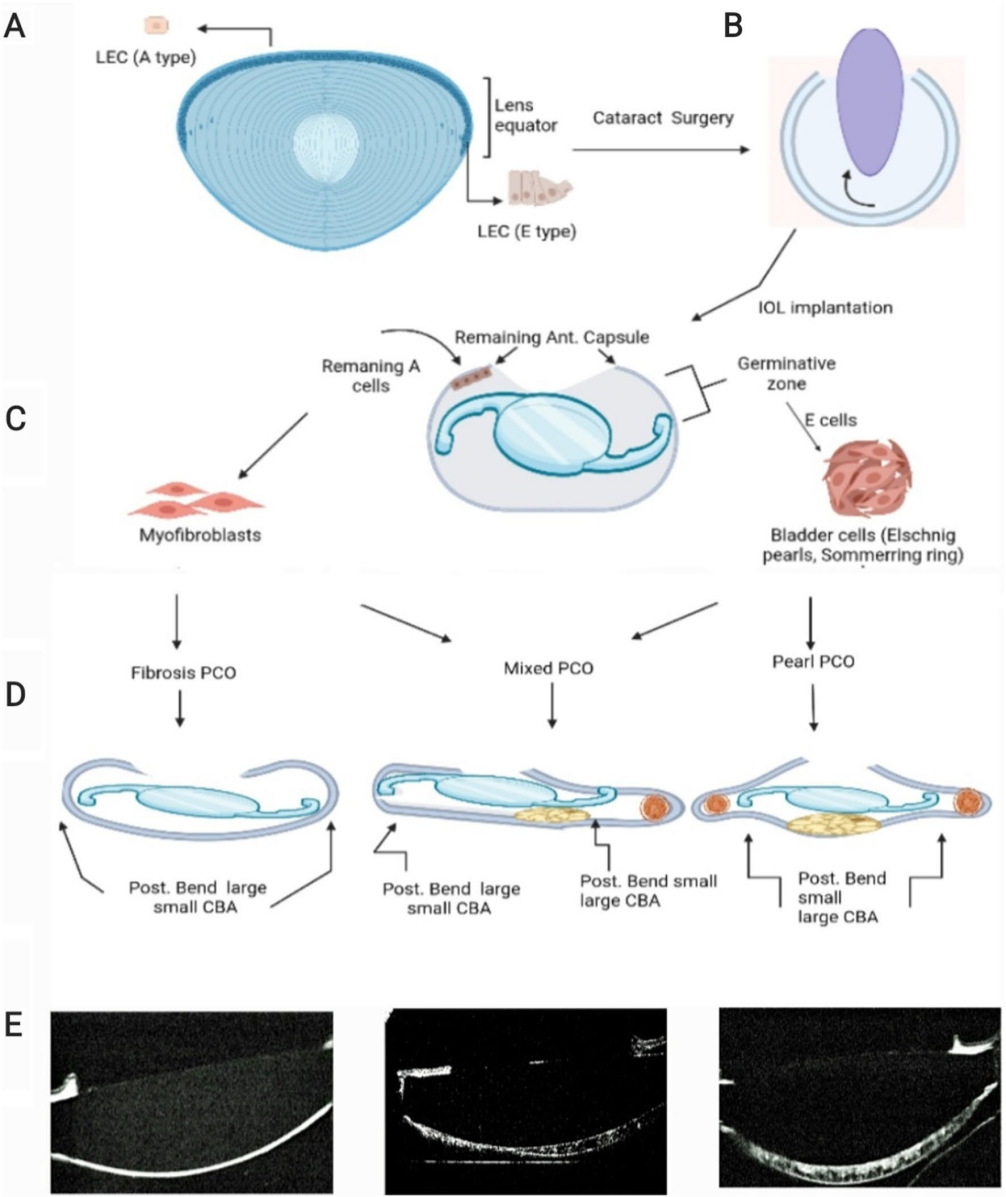
Lens structural characteristics and PCO types: **A** A schematic depiction of lens architecture displaying A and E cell types. **B** Following cataract surgery, a continuous U-shaped capsular bag is visible. **C** A discontinuous capsular bend developed following IOL implantation: The majority of active proliferation in the adult lens takes place in the germinative zone, which is immediately anterior to the lens equator. After cataract surgery, "A cells," or cells from the anterior monolayer, are thought to go through an epithelial to mesenchymal transition and become myofibroblasts. The "E cells" in the lens originate from the pre-equatorial region and frequently change into Elschnig pearls, which resemble fiber-like cells. **D** Schematic illustration of different forms of PCO growing around an IOL. **E** matching AS-OCT pictures of different types of PCO. PCO: posterior capsular opacification, LEC: Lens epithelial cells, IOL: intraocular lens. AS-OCT: anterior segment optical coherence tomography. Created with BioRender.com. http://BioRender.com

Capsular overlap on the IOL was either complete or partial, and IOL-PC adhesion was either complete or incomplete. Since LEC migration has been found to be halted at the capsular bend generated peripherally toward the capsule's equatorial zone [9], we defined the CBA as the angle formed by the PC and equatorial capsular-IOL optic edge junction. Drawn around the IOL are a pair of tangential lines that touch both of the capsule's edges. CBA was determined as the angle between (Fig. 2 and Supplementary Fig. 2). When a sheet is bent, included and complementary angles are formed [17]. The degree of bending increases as the included angle decreases, and vice versa. The angle was averaged at the superior, inferior, nasal, and temporal locations along the vertical and horizontal meridians (Supplementary Fig. 3).

## Statistical analysis

Statistical analyses were performed using IBM SPSS Statistics 28^®^ (SPSS Inc., IBM Armonk, NY, USA). Quantitative data were summarized using the mean, standard deviation, median, minimum, and maximum values, whereas categorical data were summarized using frequency (count) and relative frequency (%). To compare quantitative variables, nonparametric Kruskal–Wallis and Mann–Whitney tests were utilized. To compare categorical data, the chi-squared test was employed. The exact test was employed in its place when the expected frequency was less than 5. Correlations between quantitative variables were calculated using the Spearman' correlation coefficient. The appropriate cut-off values of important parameters for case detection were determined using area under curve (AUC) analysis and the receiver operating characteristic (ROC) curve. Statistical significance was set at *P* values less than 0.05. The Cohen’s kappa (*k*) of agreement was used to examine agreement between categorical variables, with k values less than 0.20 regarded as poor agreement and values more than 0.61 deemed good agreement.

## Results

The study enrolled 30 eyes from 24 patients: 13 males (17 eyes) and 11 females (13 eyes). The patients had an average age of 60.23 ± 11.35 years (range 35–79 years). The time between cataract surgery and current evaluation was 2.43 ± 1.48 years (range 0.25–5 years). The control group consisted of 12 eyes from 10 participants who did not have clinically significant PCO, including 6 males and 4 females. The control group participants had an average age of 64.33 ± 7.52 years (range 48–73 years) and an interval from cataract surgery of 1.96 ± 0.78 years (range 1–3 years). With *P* values of 0.179, 0.731, and 0.342, there was no statistically significant difference between the groups in terms of participant age, gender, or cataract interval. There was a statistically significant reduction in mean BCVA (decimal notation) in the PCO eyes (0.36 ± 0.20) compared to the control group (0.70 ± 0.20) (*P* > 0.001).

### PCO characterization

According to AS-OCT and biomicroscopic examination of eyes with PCO, 10 eyes (33.3%) were classified as fibrosis-type PCOs, 9 eyes (30%) as pearl-type PCOs, and 11 eyes (36.7%) as mixed-type PCOs.

**Fig. 3:**
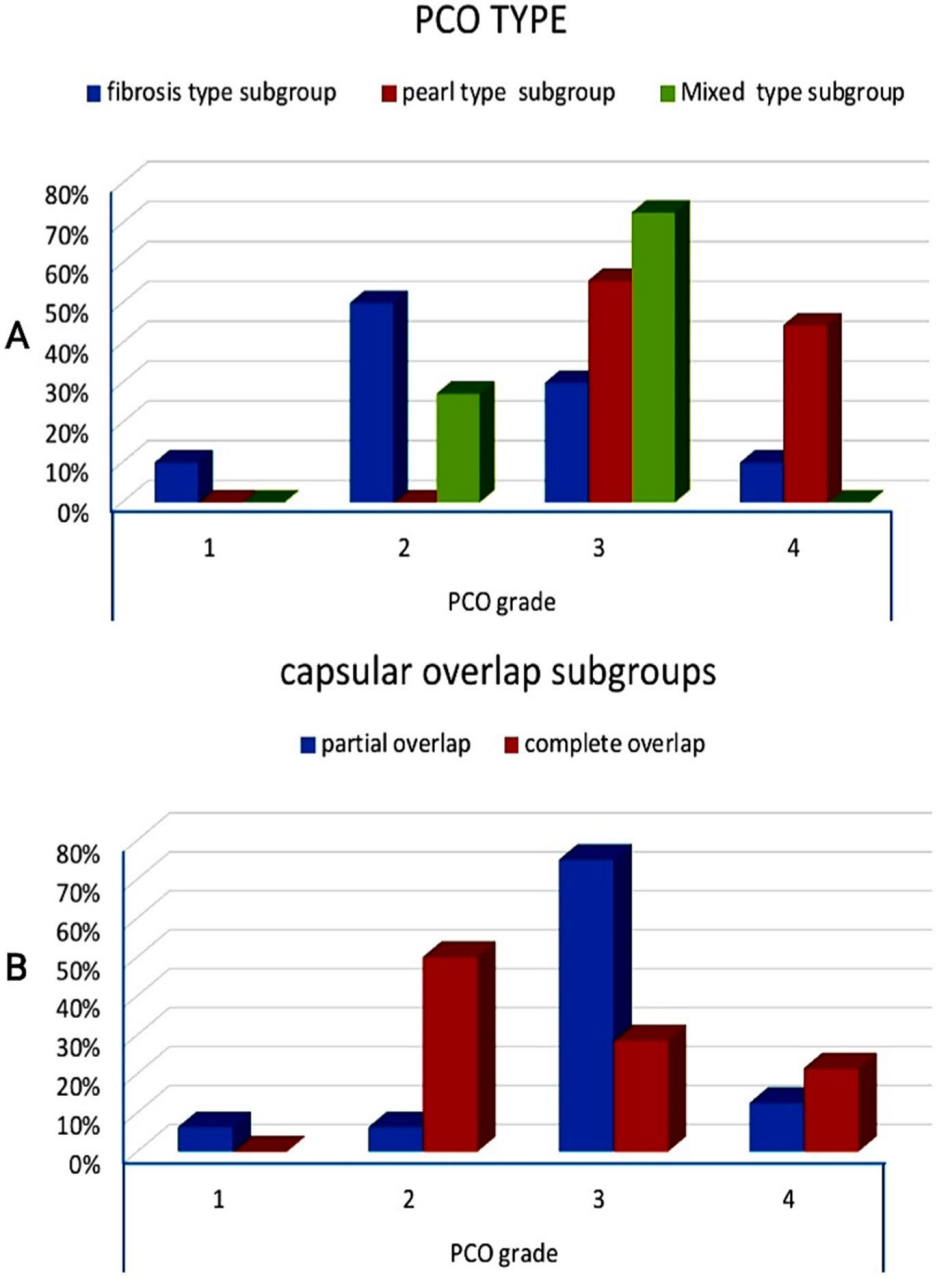
Comparative charts showing PCO grading in **A** different PCO types. **B** Capsular overlap types. PCO: posterior capsular opacification

With a p value greater than 0.001, the PCO eyes had a statistically significant increase in mean PCO area and score at the 6-, 5-, and 3-mm optic zones compared to the control group. There was a statistically significant difference in mean PCO area and score at the 6-, 5-, and 3-mm optic zones in different PCO types (*P* = 0.001), with the pearl type having the highest value, followed by the mixed type, and then the fibrosis type. However, the difference in PCO density between PCO types was statistically insignificant, with *P* values of 0.156, 0.167, and 0.093 at the 6, 5, and 3 mm optic zones, respectively (Table 1). There was a statistically significant difference in PCO grade between PCO types, with a *P* value of 0.012 (Fig. 3A).

**Table 1:** AS-OCT measurements of PCO characteristics (area, density, and score) (*n* = 42)

	Control	Fibrosis type subgroup	Pearl type subgroup	Mixed type subgroup	*P* value
PCO area at 6 mm	0.08 ± 0.06	0.28 ± 0.11	1.02 ± 0.68	0.47 ± 0.22	< 0.001
PCO density at 6 mm	60.71 ± 17.58	87.40 ± 29.73	73.67 ± 10.54	68.11 ± 20.27	0.157
PCO score at 6 mm	4.24 ± 2.68	22.56 ± 6.85	76.79 ± 53.98	30.03 ± 11.99	< 0.001
PCO area at 5 mm	0.06 ± 0.03	0.22 ± 0.09	0.90 ± 0.63	0.40 ± 0.22	0.001
PCO density at 5 mm	62.50 ± 24.72	90.78 ± 35.21	73.32 ± 10.48	68.12 ± 19.47	0.167
PCO score at 5 mm	3.64 ± 2.12	18.23 ± 7.06	67.68 ± 50.72	25.26 ± 13.18	0.001
PCO area at 3 mm	0.06 ± 0.06	0.14 ± 0.07	0.60 ± 0.43	0.26 ± 0.14	< 0.001
PCO density at 3 mm	72.98 ± 25.78	96.34 ± 35.42	77.54 ± 12.57	71.62 ± 21.35	0.093
PCO score at 3 mm	2.74 ± 1.83	11.69 ± 4.49	46.70 ± 34.51	18.04 ± 10.58	0.001

Score = area × (density − light scatter intensity), AS-OCT: anterior segment optical coherence tomography

### Capsular-IOL interactions

The differences in the mean IOL-PC distances between the PCO and control groups were statistically significant (*P* = 0.001). The results revealed that the pearl type had the greatest mean IOL-PC distance, followed by the mixed and fibrosis types, with a *P* value of 0.006 (Table 2).

**Table 2:** IOL-PC distances in studied groups (*n* = 42):

	IOL-PC distance (µm)			
	Mean ± SD	Median	Minimum	Maximum
Control	18.30 ± 5.43	18.54	11.31	30.16
Fibrosis type	45.12 ± 11.91	42.50	26.42	62.92
Pearl type	123.56 ± 61.96	106.53	53.98	205.42
Mixed type	97.45 ± 87.23	77.16	36.04	332.96

IOL-PC: intraocular lens-posterior capsule

In terms of capsular overlap and adhesion, there was a statistically significant increase in the occurrence of partial overlap and incomplete adhesion in the PCO group compared to that in the control group, with *P* values of 0.001, 0.003, respectively. With a *P* value of 0.012, there was a statistically significant increase in clinical PCO grading in the partial overlap subgroups compared to the complete overlap subgroups (Fig. 3B).

With P values of 0.592 and 0.346, respectively, there was no statistically significant difference between partial and complete overlap or complete and incomplete adhesion in different PCO types. Furthermore, there was no statistically significant difference between partial or complete overlap or complete or incomplete adhesion and PCO area, density, and score at the 6-, 5-, and 3-mm optic zones.

**Fig. 4:**
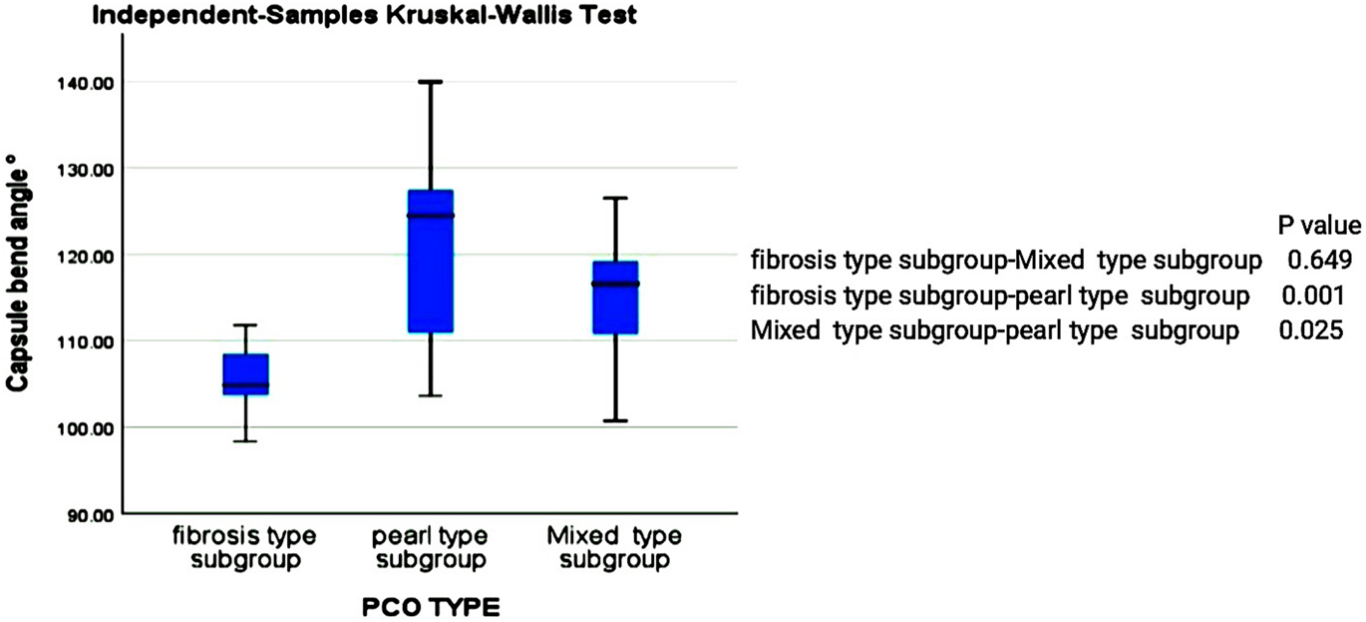
Comparison between fibrosis-type, pearl-type and mixed-type PCO regarding capsule bending angle and corresponding *P* value; A Kruskal Wallis Test was used to compare the differences. PCO: posterior capsular opacification

The PCO group had a significantly higher mean CBA than the control group (*P* = 0.001). With a *P* value of 0.003, there was also a statistically significant difference in the mean CBA between the PCO types. The pearl PCO type had the highest mean CBA, followed by mixed and fibrosis (Table 3, Fig. 4). CBA's mean in the partial overlap subgroup was 115.84 with a standard deviation of 8.52, while it was 110.79 + 10.91 in the complete overlap subgroup (*P* value = 0.313, negligible).

**Table 3:** Capsule bending angle in studied groups (*n* = 42)

	Capsule bending angle°			
	Mean ± SD	Median	Minimum	Maximum
Control	91.08 ± 3.18	91.50	84.90	95.40
Fibrosis type	105.57 ± 3.94	104.88	98.30	111.80
Pearl type	120.48 ± 11.76	124.50	103.60	140.00
Mixed type	114.96 ± 6.99	116.60	100.70	126.50

**Fig. 5:**
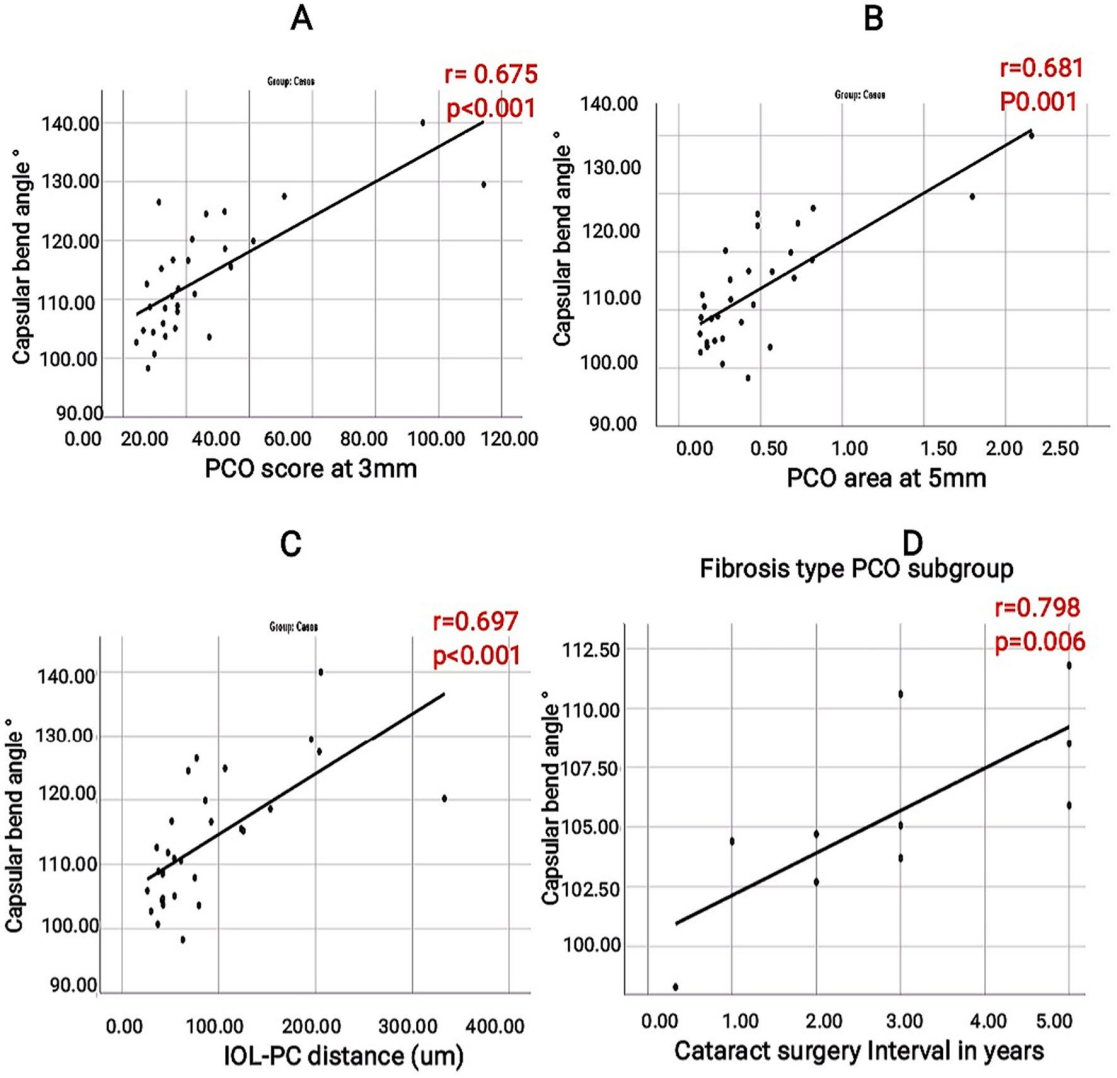
Scatter plot of Capsular Bend degree versus PCO score at 3 mm **A**, PCO area at 5 mm **B**, IOL-PC distance **C**, and cataract surgery timing in fibrosis type PCO **D**. PCO: Posterior Capsular Opacification. IOL-PC: Intraocular lens-posterior capsule

CBA had a statistically significant positive correlation with IOL-PC distance, PCO area, and score at the 6-, 5-, and 3-mm optic zones (*P* = 0.001). In fibrosis-type PCO, there was a statistically significant positive correlation between CBA and cataract surgery interval, with a *P* value of 0.006 (Table 4, Fig. 5).

**Table 4:** Correlation between Capsular bending angle and PCO characterization in PCO cases (*n* = 30)

	Cases		
	Capsule bending angle		
	Correlation Coefficient	*P* value	*N*
IOL-PC distance (µm)	0.697	< 0.001	30
PCO area at 6 mm	0.676	< 0.001	30
PCO density at 6 mm	−0.132-	0.487	30
PCO score at 6 mm	0.665	< 0.001	30
PCO area at 5 mm	0.681	< 0.001	30
PCO density at 5 mm	−0.079-	0.677	30
PCO score at 5 mm	0.685	< 0.001	30
PCO area at 3 mm	0.653	< 0.001	30
PCO density at 3 mm	−0.043-	0.820	30
PCO score at 3 mm	0.675	< 0.001	30

There was a positive connection between CBA and IOL-PC distance, PCO area, and score at 6-, 5-, and 3-mm optic zones, with *P* values of 0.009, 0.004, 0.001, 0.009, 0.004, 0.009, and 0.001 in complete overlap subgroups, respectively. Additionally, in partial overlap subgroups, there was a positive connection between CBA and IOL-PC distance, PCO area, and score at 6-, 5-, 3-mm optic zones (*P* values = 0.009, 0.013, 0.026, 0.010, 0.015, 0.029, and 0.039, respectively).

### Receiver operating characteristic (ROC) curve analysis

**Fig. 6:**
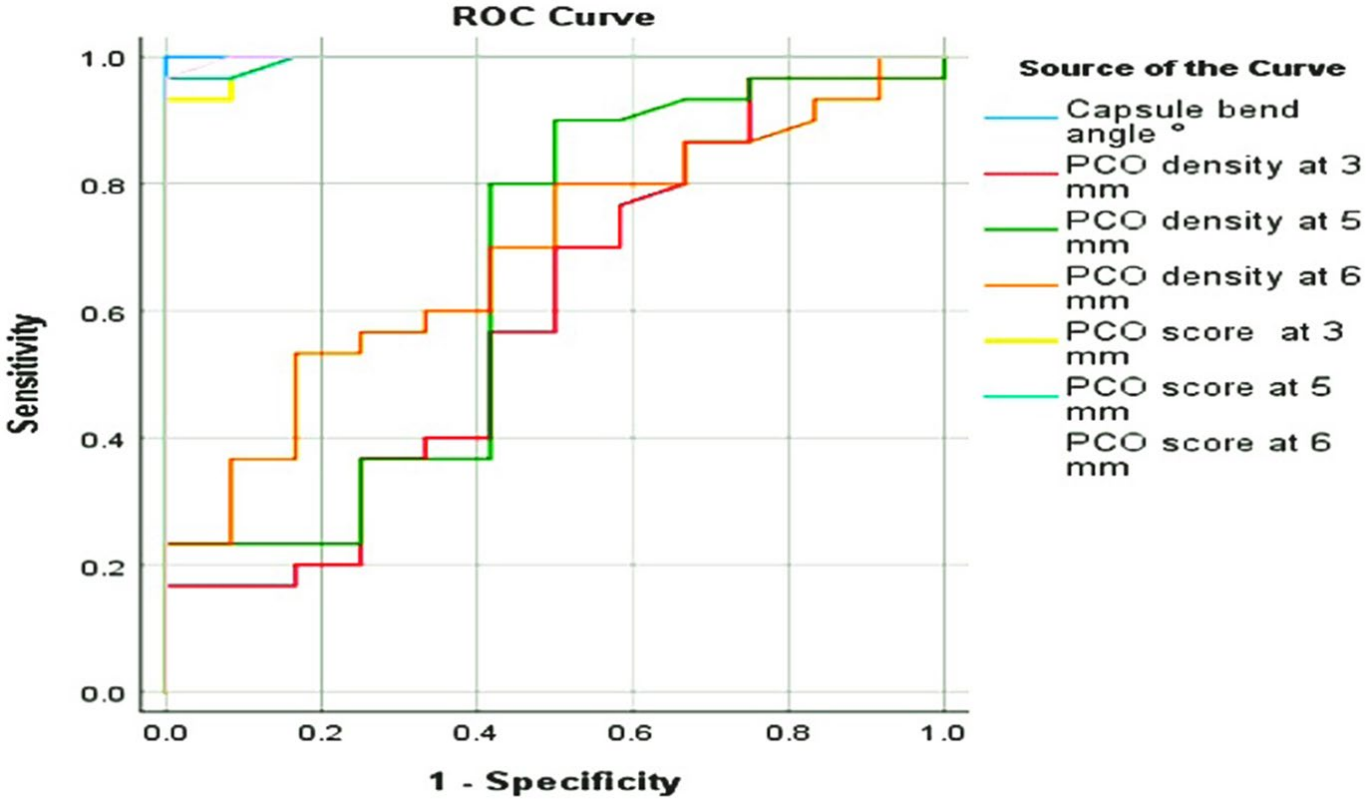
Receiver-operating characteristic (ROC) curves analysis between cases and control regarding capsule bending angle, PCO score and density at 3, 5, and 6 distances. The inset reports the different areas under the curve (AUC) and the corresponding significance values with an AUC value of 0.5

In terms of CBA, the cut-off value for the ROC curve for distinguishing between PCO patients and controls was 96.85°. The CBA showed complete specificity and sensitivity. The ROC curve output data for PCO score discrimination power at the 6-, 5-, and 3-mm optic zones to differentiate between PCO cases and controls revealed cut-off values of 10.75, 8.905, and 7.27, respectively (Fig. 6). The ROC curve output data for the discrimination power of the degree of capsular overlap to detect CBA and PCO density and score at the 6-, 5-, and 3-mm optic zones were statistically insignificant (*P* = 0.062, 0.411, 0.356, 0.212, 0.236, 0.297, 0.239, and 0.245, respectively).

### Agreement

In all eyes analyzed, the agreement between the total overlap and both CBA, PCO grade, and IOL-PC adhesion was examined. For the entire overlap and CBA, 10 eyes (41.7%) achieved positive results (*k* = 0.282; 61.9% agreement). 21 eyes (77.8%) showed complete overlap and IOL-PC adhesion (*k* = 0.438; 73.8% agreement). 7 eyes (29.2%) exhibited total overlap and a positive PCO grade (*k* = − 0.476; agreement: 26.2%). The best agreement was 73.8% in 31 eyes with total overlap and complete IOL-PC, and there was essentially no agreement between complete overlap and a positive PCO grade (Table 5). Concerning the agreement between partial overlap and CBA, PCO grade, and IOL-PC adhesion in all eyes studied: 14 (77.8%) of the eyes had partial overlap and a positive PCO grade (*k* = 0.476; 73.8% agreement). Two eyes (37.5%) had positive partial overlap and positive CBA findings (*k* = − 0.319; 38.09% agreement). Six eyes (37.5%) showed partial overlap and total IOL-PC adhesion (*k* = − 0.282; 26.19% agreement). The best agreement was 73.8% in 31 eyes with partial overlap and a positive PCO grade, and there was essentially no agreement between total overlap with both positive CBA and complete IOL-PC adhesion (Table 6).

**Table 5:** Agreement between complete overlap and other parameters: (*n* = 42)

		Complete overlap				Cohen’s k	% of agreement
		Positive		Negative			
		Count	%	Count	%		
CBA	Positive	10	41.7	2	11.1	0.282	61.9
	Negative	14	58.3	16	88.9		
PCO grade	Positive	7	29.2	14	77.8	− 0.476	26.2
	Negative	17	70.8	4	22.2		
IOL-pc adhesion	Positive	21	77.8	5	33.3	0.438	73.8
	Negative	6	22.2	10	66.7		

Cohen’s k: 0.01–0.20 slight agreement, 0.21–0.40 fair agreement, 0.41–0.60 moderate agreement, 0.61–0.80 substantial agreement, 0.81–1.00 almost perfect or perfect agreement. CBA: Capsular bend angle, PCO: Posterior capsular opacification, IOL-PC: intraocular lens- posterior capsule

**Table 6:** Agreement between partial overlap and other parameters: (*n* = 42)

		Partial overlap				Cohen’s k	% of agreement
		Positive		Negative			
		Count	%	Count	%		
CBA	Positive	2	11.1	10	41.7	− 0.319	38.09
	Negative	16	88.9	14	58.3		
PCO grade	Positive	14	70.8	7	22.2	0.476	73.8
	Negative	4	29.2	17	77.8		
IOL-pc adhesion	Positive	6	37.5	21	80.8	− 0.382	26.19
	Negative	10	62.5	5	19.2		

Cohen’s k: 0.01–0.20 slight agreement, 0.21–0.40 fair agreement, 0.41–0.60 moderate agreement, 0.61–0.80 substantial agreement, 0.81–1.00 almost perfect or perfect agreement. CBA: Capsular bend angle, PCO Posterior capsular opacification, IOL-PC intraocular lens- posterior capsule

Positive bend is defined as CBA below 96.85° (the cut-off value for control) in the positive versus. negative bend spectrum. The PCO grading grades of 2 and 3 were seen favorably. IOLs completely attached to the capsule are a sign of positive IOL-PC adhesion.

## Discussion

PCO is still the most common complication following cataract surgery with IOL implantation [18]. Despite the thinness of the lens capsule (20 µm in the anterior and 5 µm in the PC), the healing-opacified capsule that holds the IOL is fairly thick. A buildup of fibrous tissue on the inner surface of the capsule during the course of the prolonged healing phase may cause the central region of the remaining PC of the crystalline lens housing the IOL to become opaque [5].

LEC migration was inhibited at the sharp, "discontinuous" capsular bend created by the sharp edge of the optic [9]. The discontinuous capsular bend at the posterior optic edge, however, is formed only when the optic is circumferentially overlapped by the anterior capsule leaf, which is not always the case. In addition, LEC migrate posteriorly for 1–2 weeks after surgery, while the capsular bend forms in 2–4 weeks, implying that some LEC can migrate posteriorly over the capsular bend before it forms, causing later PCO. The subsequent migration of LEC stops once a bend is formed [19]. LEC at the capsular bend were found to be in the G0 phase of the cell cycle, indicating that they were contact inhibited [16], whereas other LEC proliferated, forming a Soemmering's ring. This padded, increasingly stuffed after cataract can break up the capsular bend years after surgery by spreading the capsular bag, and it may awaken contact-inhibited LEC from the G0 phase at the bend, which then begin to re-proliferate and migrate posteriorly. This could explain why the most commonly used hydrophobic acrylic IOL has a high 10-year cumulative rate of laser capsulotomy [20].

In the current study, we used AS-OCT to characterize PCO (type, density, and score). We identified capsular-IOL interactions (overlap, IOL-PC distance and adhesions, and capsular bend formation) and included CBA in the analysis of capsular-IOL interactions. We investigated the relationships between CBA and PCO as well as the effect of PCO types and CBA on the IOL-PC distance. There were three basic PCO types: fibrosis, pearl, and mixed.

We included 30 eyes with clinically significant PCO and a control group of 12 eyes without significant PCO after uneventful cataract surgery. In terms of PCO characterization, there was a statistically significant increase in the mean PCO area and score in PCO eyes compared to controls, as well as a significant difference between PCO types, with the pearl type having the highest value, followed by the mixed type, and finally the fibrosis type. However, in terms of PCO density, the fibrosis type was higher than the pearl and mixed types, but this difference was not statistically significant between the groups. According to Yu et al. [16], the area and thickness of the pear-type PCO were obviously greater than those of the fibrosis-type PCO, but the density of the pearl-type PCO was lower. However, visual acuity was found to be significantly correlated with posterior capsular thickening rather than density [12].

In the current study, the mean IOL-PC distance was significantly greater in the PCO group than in the control group. The pearl type had the greatest mean IOL-PC distance, followed by the mixed and the fibrosis types. This finding is consistent with that of Moreno-Montaes et al. [12]. Our findings are also consistent with those of Hawlina et al. [21], who found that the PC was in close contact with the IOL in control eyes, with a median IOL-PC distance of 11.5 m. They also compared the IOL-PC distances of fibrosis-type PCO to the IOL-PC distances measured in the control group. The difference was statistically significant (*P* = 0.02).

In terms of capsular bend, because the posterior bend was more important in preventing LEC migration, we considered the CBA that formed between the periphery of the IOL optics and the PC. The previous studies have demonstrated the significance of the anterior capsular bend [7, 22]. The anterior capsular bend does not prevent LEC proliferation in the equatorial region, which is responsible for the pearl-type PCO, as explained by the formation of Soemmering's ring. A posterior bend is one that inhabits and prevents LEC migration to PC beyond the bend. Even though it might be crucial in preventing LEC migration lining the anterior capsule, which results in PCO of the fibrosis type, the anterior bend does not immediately form during cataract surgery. A capsular bend could prevent further migration. As a result, it appeared that the bend formed at the PC was more important than the bend formed at the anterior capsule.

LEC that transform into cells that resemble myofibroblasts cause fibrotic PCO and can be recognized by the presence of α-smooth muscle actin (*α*-SMA), a marker for myofibroblast-like cells. In some LEC that differentiate into lens fiber cells and do not test positive for *α*-SMA, PCOs of the Soemmering ring and Elschnig's pearl types are produced [23]. According to studies by Kurosaka et al., the behaviors of the LEC that were positive for α-SMA were inconsistent, and some of them vanished, leaving cell-free areas on the posterior capsule. On the other hand, LEC that were α-SMA-negative migrated from the Soemmering ring and also differentiated on the central posterior capsule beneath the IOL optic to create the Elschnig's pearls type of PCO [24].

The PCO group had a significantly higher mean CBA than the control group (*P* = 0.001). With a *P* value of 0.003, there was also a statistically significant difference in the mean CBA between the PCO types. The pearl PCO type had the highest mean CBA, followed by the mixed and fibrosis types. These findings are in favor that pearl-type PCO results from equatorial LEC migration, resulting in bag distension, increased CBA, and therefore a decreased capsule bending degree. In pearl-type PCO, the bend may be observed around the haptic, but at other points around the optic, it may be difficult to recognize. However, it appears that in PCO of the fibrosis type, the angle is stabilized by the fibrosis. With increasing intervals of cataract surgery timing, there was a statistically significant increase in CBA in eyes with fibrosis-type PCO. This finding may reflect the dynamic nature of the capsular bend, and increasing the angle may disrupt LEC migration inhibition.

Fang et al. reported two types of capsular bends: complete adhesion and incomplete adhesion. They classified incomplete adhesion into three types: funnel adhesion, parallel adhesion, and detached adhesion. The incomplete adhesion index (IAI) ranged from 0 to 1 and varied between eyes. Both the PCO score and area were significantly higher in the high IAI group (greater than 0.50) than in the low IAI group (*P* = 0.05). Furthermore, the PCO score and area were significantly higher in the cohort with at least one IA-D capsular bend in six districts than in the control group (*P* = 0.05). They proposed that the type of capsular bend could be used as an index to predict PCO [22].

Our findings showed that there was a statistically significant difference in PCO grades between PCO types. High grades were noted in the pearl type, followed by the mixed type, and finally the fibrosis type (*P* = 0.012). These results show that AS-OCT PCO scoring is associated with clinical PCO grading. According to Lu et al. [25], the pear-type PCO had a larger opacification area in the IOL-posterior capsule space at 6-, 5-, and 3-mm optic zones and was thicker than the fibrosis PCO. Furthermore, objective assessment of vision quality using the Optical Quality Analysis System III revealed that the objective scatter index was significantly higher in pear-type PCO than in fibrosis-PCO (Z = 4.06, *P* = 0.001).

Total overlap displayed the best agreement with the complete IOL-PC adhesion type in the current investigation, with 73.8% in 31 eyes, followed by CBA by 61.9%. However, partial overlap exhibited a 73.8% agreement with a positive PCO grade. There was no statistically significant difference between partial and complete overlap or complete and incomplete adhesion in the different PCO types (P = 0.592 and 0.346, respectively). Furthermore, the PCO area, density, and score at the 6-, 5-, and 3-mm optic zones did not differ significantly depending on whether there was partial versus complete overlap or complete versus incomplete adhesion. Fang et al.'s analysis of the PCO score and area found no statistically significant differences between eyes with complete and incomplete adhesion, whereas the partial overlap group showed a statistical difference greater than the complete overlap group (*P* = 0.05) [22].

The ROC curve output data for the discrimination power of CBA between PCO and control eyes revealed that the cut-off value was 96.85°. These findings support the idea that PCO is reduced by increasing the degree of capsular bend. Capsular bends reduce PCO formation by preventing LEC migration, but they can be overcome by capsular destinations. It appears that capsular bend has a limit in terms of preventing PCO formation.

The current study discovered that CBA had a statistically significant positive link with IOL-PC distance, PCO area, and score at the 6-, 5-, and 3-mm optic zones (*P* = 0.001). These implications imply that reducing CBA by increasing the degree of capsular bend is linked to a reduction in IOL-PC space and would favor a decrease in PCO development, particularly pearl-type PCO. According to research by Pallikaris et al., a hydrophilic acrylic ring placed inside the eye prior to the IOL creates an open capsule environment that allows for a quick and secure IOL insertion as well as postoperative stabilization. Additionally, no PCO was found (59 patients with a one-year follow-up and 120 cases with a six-month follow-up). The ring acts as a mechanical barrier to stop PCO [26]. This work offers a real-world application of the importance of LEC separation and capsular bend development.

The limitations of the current study include a relatively small sample size. While calculating the degree of capsular bend, we employed the included angle rather than the complementary angle. The complementary angle is always used in the bend allowance calculation, albeit this is not mentioned in the current study. We did not morphologically classify capsular bends.

## Conclusions

The degree of capsular bend and CBA had a statistically significant relationship with the IOL-PC distance as well as the PCO area and score. AS-OCT can be used as an additional tool for PCO characterization in clinical studies. Capsular bends reduce PCO formation by preventing LEC migration, but they can be broken by the capsular destination.

### Supplementary Information

Below is the link to the electronic supplementary material.

Supplementary file1 (JPG 91 KB)

Supplementary file2 (JPG 122 KB)

Supplementary file3 (JPG 105 KB)

Supplementary file4 (DOCX 18 KB)

## Data Availability

This published article includes all data generated or analyzed during the current study.

## Abbreviations

*α*-SMA: *α*-Smooth muscle actin
µm: Micrometer
mm: Millimeter
AS-OCT: Anterior segment optical coherence tomography
AUC: Area under curve
BCVA: Best corrected visual acuity
CBA: Capsule bending angle
IAI: Incomplete adhesion index
IOL: Intraocular lens
LEC: Lens epithelial cells
Nd-YAG: Neodymium doped-yttrium aluminum garnet
OCT: Optical coherence tomography
PC: Posterior capsule
PCO: Posterior capsular opacification
ROC: Receiver operating characteristic
