# Supplementary figures and images for: Analysis of the capsular bend in posterior capsular opacification using anterior segment optical coherence tomography
Source: Int Ophthalmol. 2023 Oct 28;43(12):4945–58. doi: 10.1007/s10792-023-02897-7 (PMC10724338; doi:10.1007/s10792-023-02897-7)

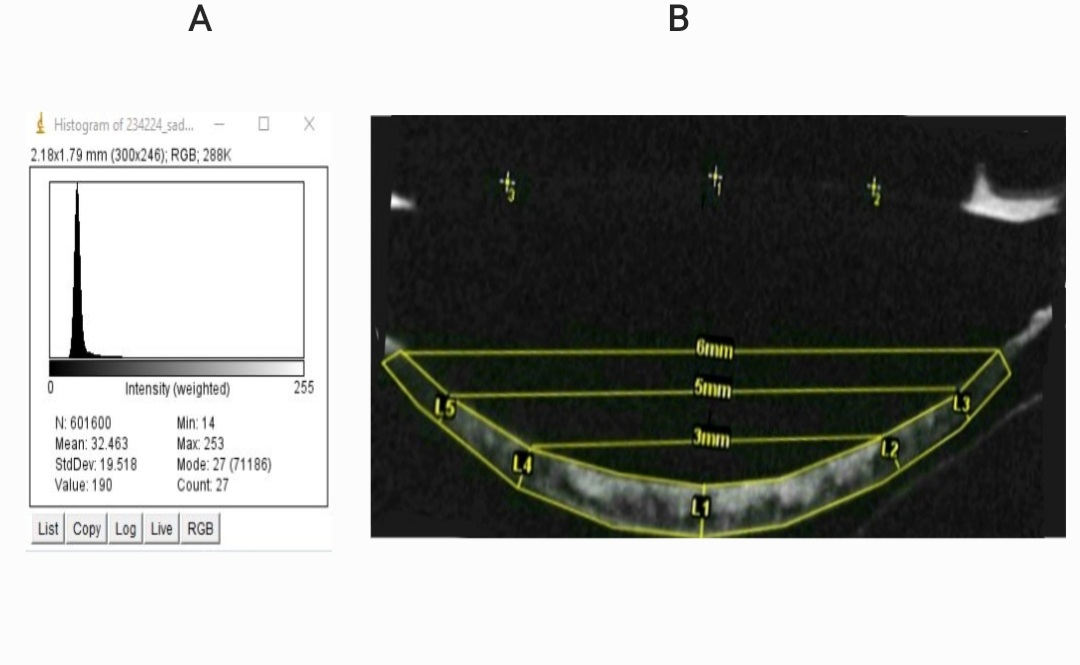

Supplement: Supplementary file 1 — Supplementary file1 (JPG 91 KB) [file 10792_2023_2897_MOESM1_ESM.jpg]

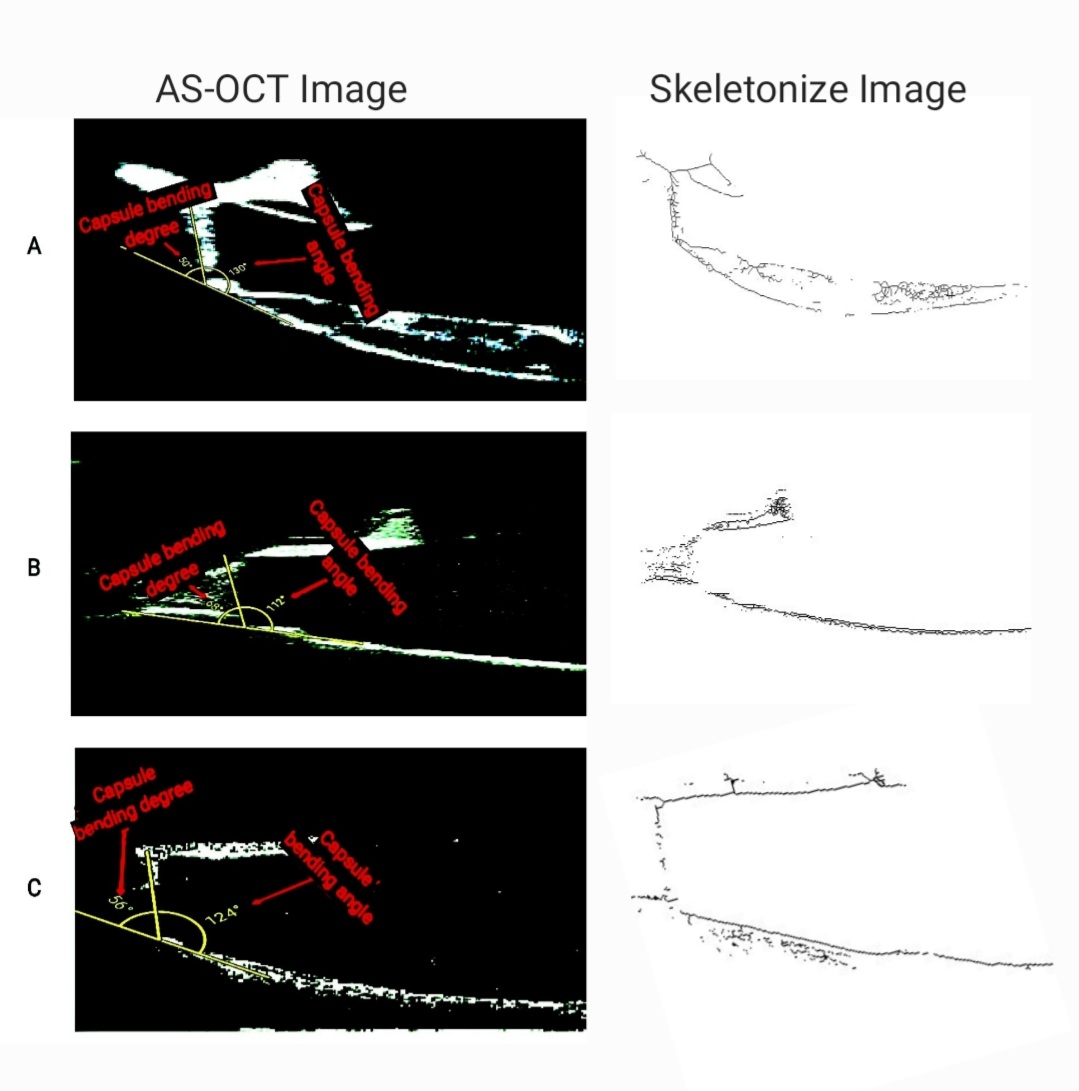

Supplement: Supplementary file 2 — Supplementary file2 (JPG 122 KB) [file 10792_2023_2897_MOESM2_ESM.jpg]

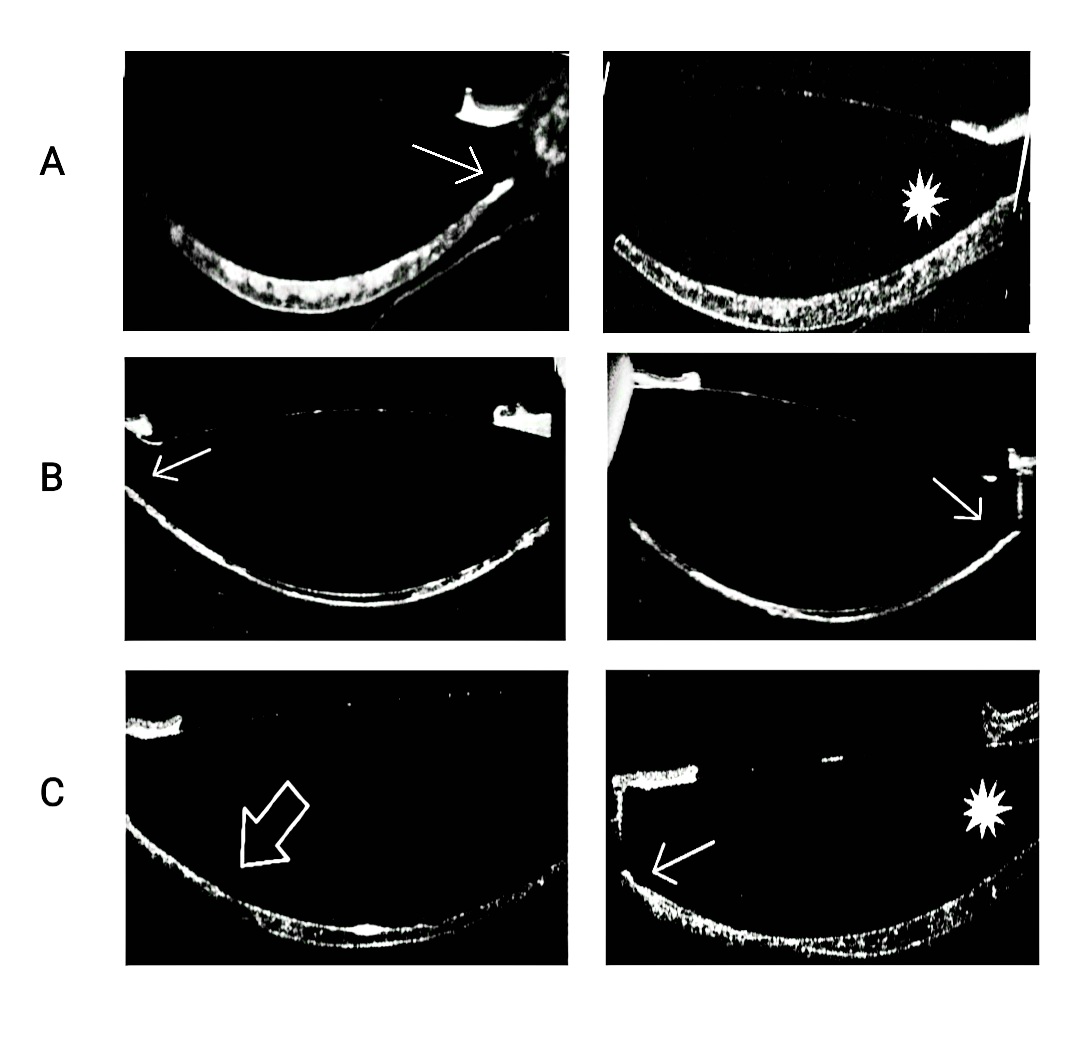

Supplement: Supplementary file 3 — Supplementary file3 (JPG 105 KB) [file 10792_2023_2897_MOESM3_ESM.jpg]
