# Supplementary material for: Analysis of the capsular bend in posterior capsular opacification using anterior segment optical coherence tomography
Source: Int Ophthalmol. 2023 Oct 28;43(12):4945–58. doi: 10.1007/s10792-023-02897-7 (PMC10724338; doi:10.1007/s10792-023-02897-7)
Supplement: Supplementary file 4 — Supplementary file4 (DOCX 18 KB) [file 10792_2023_2897_MOESM4_ESM.docx]

**Supplementary Figures legends**

**Supplementary Figure (1):** Composite image of the PCO evaluation method using Image J software. (A) Histogram of the image intensity showing mean density 32.5. (B) Illustration graph of RTVue-100 OCT measurement of PCO characteristics in one cross-sectional image (the figure was acquired at the horizontal meridian). The lengths of L1, L2, L3, L4, and L5 represent PCO thicknesses at the point of the central optic region and at the 3 mm and 5 mm diameter of the IOL optic region. PCO: posterior capsular opacification, OCT: optical coherence tomography.

**Supplementary Figure (2):** Images depicting measures of capsular bending angle and degree in various PCO types using AS-OCT images and matching skeletonized images generated by Image J software; (A) Pearl-type PCO; (B) fibrosis-type PCO; (C) mixed-type PCO. AS-OCT: anterior segment optical coherence tomography, PCO: posterior capsular opacification, CBA: capsule bending angle.

**Supplementary Figure (3)**: AS-OCT images demonstrating varied capsular bend performance in different PCO types. A) Pearl-type PCO with capsular overlap and bend development around the haptic (Arrow), but poorly identifiable distant from the haptic (Asterisk). B) Fibrosis-type PCO with capsular overlap (detachment type), bend development in various regions (Arrows).C) Mixed-type PCO with capsular overlap (funnel type), bend formation in fibrosis area (arrow), but poorly identifiable away (Asterisk), and fibrosis stops progression of Elschnig's pearls on the posterior capsule (Hollow Arrow).
